# Morphometric Similarity Patterning of Amyloid-*β* and Tau Proteins Correlates with Transcriptomics in the Alzheimer’s Disease Continuum

**DOI:** 10.3390/ijms252312871

**Published:** 2024-11-29

**Authors:** Lorenza Brusini, Giorgio Dolci, Lorenzo Pini, Federica Cruciani, Fabrizio Pizzagalli, Paolo Provero, Gloria Menegaz, Ilaria Boscolo Galazzo

**Affiliations:** 1 Department of Engineering for Innovation Medicine, University of Verona, 37134 Verona, Italy; 2 Department of Computer Science, University of Verona, 37134 Verona, Italy; 3 Department of Neuroscience, University of Padova, 35121 Padova, Italy; 4 Istituto Fondazione Oncologia Molecolare Ente del Terzo Settore (IFOM ETS)–The Associazione Italiana per la Ricerca sul Cancro (AIRC) Institute of Molecular Oncology, 20139 Milano, Italy; 5 Department of Neurosciences “Rita Levi Montalcini”, University of Turin, 10126 Turin, Italy

**Keywords:** T1-weighted MRI, diffusion MRI, ADNI, partial least squares, gene expression, enrichment analysis

## Abstract

Bridging the gap between cortical morphometric remodeling and gene expression can help to clarify the effects of the selective brain accumulation of Amyloid-β (Aβ) and tau proteins occurring in the Alzheimer’s disease (AD). To this aim, we derived morphometric similarity (MS) networks from 126 Aβ- and tau-positive (Aβ+/tau+) and 172 Aβ−/tau− subjects, and we investigated the association between group-wise regional MS differences and transcriptional correlates thanks to an imaging transcriptomics approach grounded in the Allen Human Brain Atlas (AHBA). The expressed gene with the highest correlation with MS alterations was *BCHE*, a gene related to Aβ homeostasis. In addition, notably, among the most promising results derived from the enrichment analysis, we found the immune response to be a biological process and astrocytes, microglia, and oligodendrocyte precursors for the cell types. In summary, by relating cortical MS and AHBA-derived transcriptomics, we were able to retrieve findings suggesting the biological mechanisms underlying the Aβ- and tau- induced cortical MS alterations in the AD continuum.

## 1. Introduction

Imaging techniques enabling the possibility to observe, in vivo, the prints of the biological processes acting on the brain are of extreme importance. In particular, finding imaging markers able to reflect specific pathological mechanisms is valuable especially in clinics. Positron emission tomography (PET) has enabled the detection of Amyloid-β (Aβ) and tau protein accumulation, the molecular hallmarks of Alzheimer’s disease (AD), demonstrating that Aβ plaques and neurofibrillary tangles formed from phosphorylated tau protein can be detected several years before the onset of clinical symptoms [1]. Consequently, the risk of developing AD or an early diagnosis can potentially be identified during pre-clinical stages, allowing for timely intervention through therapies aimed at preventing, slowing, or even halting disease progression.

In addition to being valuable in aiding clinical practice and drug development, in vivo imaging markers sensitive enough to detect changes in the AD continuum would also be relevant in understanding its pathophysiology. Indeed, a comprehensive picture of alterations in AD is essential to understand the complexity of modifications occurring during the disease. Vilkaite et al. [2] illustrated the potential of linking omics data (genomics, proteomics, and transcriptomics) to molecular imaging to uncover the mechanisms relating Aβ and tau in AD. Omics information can be related to different scales of imaging and not ultimately standard non-invasive in vivo magnetic resonance imaging (MRI) of the whole brain. By taking advantage of the Allen Human Brain Atlas (AHBA) and PET-MRI with different tracers to distinctively capture Aβ and tau, Yu and colleagues [3] were able to confirm the existence of a specific sensibility to these two AD hallmarks depending on the spatial location on the brain. In fact, AHBA provides the gene expression profiles for 20,736 genes encoding proteins for each brain region, thus enabling the discovery of the possible relationships between genetics and imaging at a higher spatial resolution. Studying the imaging transcriptomics of these pathological mechanisms with other MRI modalities may bring to light novel insights for the understanding of AD.

To the best of our knowledge, works attempting to investigate the imaging transcriptomics relationship to explain the role and behaviour of Aβ and tau in AD mainly rely on PET [2,3,4,5,6]. On top of this, no work exists on imaging transcriptomics for studying Aβ and tau in the AD continuum based on cortical morphometric similarity (MS). Typically, in vivo morphometric features of the brain tissue are derived from the conventional T1-weighted (T1w) MRI. Cortical volume, surface area, and thickness are the most known, and they have been extensively used in the literature for characterizing brain variations in both healthy and pathological individuals. T1w-MRI in AD is proven to be able to show signs of the atrophy encountered by patients along with the disease’s progression [7], generally in the late stage. While T1w-MRI captures macroscopic characteristics of the brain tissue, diffusion (d)MRI, instead targeting the diffusion of water molecules, can indirectly reveal microscopic insights about geometry, architecture, and topology [8]. Recent studies have shown that microstructural abnormalities in gray matter can be detected in cognitively unimpaired (CN) or mild cognitively impaired (MCI) individuals depending on the Aβ and/or tau positivity [9,10]. Moreover, Spotorno et al. [9] demonstrated that the dMRI-derived microscopic alterations occurred before brain atrophy arises, evidenced by T1w-MRI. Thus, relying on features derived from both T1w-MRI and dMRI, an MS network (MSN) representing patterns of similarity between regions can be constructed at a single subject’s level. The biological meaning of the MSN is grounded in evidence that cortical areas showing a similar cytoarchitecture are probably more anatomically connected [11,12].

Linking MSN and transcriptomics has already been proven to be able to boost research in several fields. Through such an approach, Morgan et al. [13] shed light on the biological mechanisms underlying schizophrenia, showing that MS was reduced in patients compared to controls and highlighting that the pattern was associated with the expression of genes enriched for processes including the development of the nervous system and synaptic signaling. In [14], Li et al. studied the biological mechanisms relating genetics with MS alterations in generalized epilepsy, while Lei and colleagues [15] and Martins et al. [16] studied those in pediatric bipolar disorder and in chronic pain. Generally, the method also proved to be effective in contributing to knowledge on the advancement of post-traumatic stress disorder [17], Parkinson’s disease [18], and COVID-19 [19]. Notably, only one work attempted to do such an investigation in AD [20]. In detail, Zhang et al. compared MS between advanced AD patients and healthy subjects. The differences found included an increased MS in the frontal cortex and occipital cortex and a decreased MS in the temporal and parietal cortex. The authors also found an association between these alterations and memory function, and among the biological processes enriched, the most significant term was related to synaptic transmission.

To summarize, for the first time, with the present work, we aim at characterizing cortical MS on the AD continuum by developing MSNs relying on both macro- and microscopic features of the brain tissue of individuals from the pre-clinical to the MCI stage, categorized as Aβ-positive/tau-positive (Aβ+/tau+) and Aβ-negative/tau-negative (Aβ−/tau−). Furthermore, by establishing the link with transcriptomics, we will reach our second aim of finding the viable biological mechanisms underlying these two processes (i.e., Aβ and tau).

The categorizations Aβ+/tau+ and Aβ−/tau− do not consider more complex cases (Aβ+/tau− and Aβ−/tau+), but they maximize the effects due to the presence of the AD hallmarks. Moreover, such a strategy will also maximize the probability of investigating cortical MS patterning due to the effects of Aβ and tau in the specific pre-clinical condition of AD.

## 2. Results

### 2.1. Regional Morphometric Similarity Group-Wise Differences

**Figure 1:**
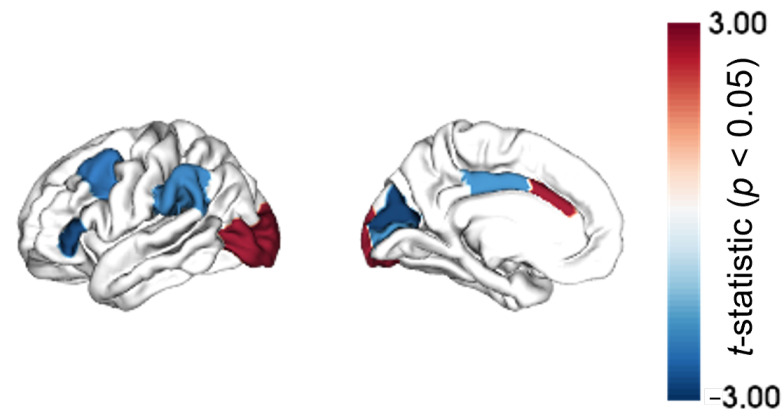
Differences in regional morphometric similarity between Aβ+/tau+ and Aβ−/tau− represented on cortical maps and quantified as *t*-statistics. Only brain regions with a *p*-value < 0.05 are highlighted. Note: No region was retained after false discovery rate correction.

Figure 1 shows the differences in regional MS between Aβ+/tau+ and Aβ−/tau− groups. More precisely, eight out of thirty-four brain regions had a significant regional MS difference (*p* < 0.05, uncorrected for multiple comparisons). Regional MS was increased in Aβ+/tau+ compared to Aβ−/tau− in the caudal anterior cingulate and in the lateral occipital cortex, suggesting an increased similarity with the other brain areas in terms of morphometric and microstructural features. Conversely, MS decreased in Aβ+/tau+ compared to Aβ−/tau− in the caudal middle frontal cortex, in the cuneus, in the pars triangularis, in the pericalcarine, in the posterior cingulate, and in the supramarginal cortex. No region was retained after multiple comparisons correction with false discovery rate (FDR), although $p_{FDR}$ was close to significance ($p_{FDR}$ = 0.07) in the caudal anterior cingulate, in the lateral occipital cortex, in the cuneus, and in the pars triangularis.

### 2.2. Regional Morphometric Similarity and Transcriptomics Relationship

**Figure 2:**
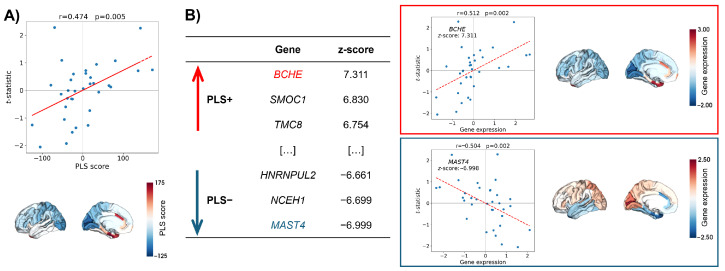
Transcriptomic profiles related to differences in morphometric similarity between Aβ+/tau+ and Aβ−/tau−. (**A**) Weighted gene expression profiles calculated as partial least squares (PLS) regression-derived scores shown (i) in association with the regional MS group-wise differences in the scatter plot on the upper part of the panel and (ii) on cortical maps on the lower one. (**B**) The left part of the panel shows the table summarizing the top three genes and related *z*-scores positively and negatively impacting the PLS regression, respectively; the right part of the panel, instead, shows the scatter plot with gene expression profiles versus regional MS group-wise differences and gene expression-related cortical maps for both the most positively (*BCHE*, at the top) and the most negatively (*MAST4*, at the bottom) impactful genes.

The association between regional MS changes in Aβ+/tau+ versus Aβ−/tau− and brain transcriptomics profiles as investigated with partial least squares (PLS) was deemed significant by the permutation test (*p* = 0.039). The PLS scores related to the weighted gene expression values were positively correlated with the differences in regional MS expressed as t-statistics (*r* = 0.474, *p* = 0.005) (Figure 2, panel A). Such a relationship translates for a high expression of genes with a positive PLS weight in regions where MS was increased, and conversely, genes with a negative PLS weight were highly expressed in regions where MS decreased.

The gene ranking was based on significant *z*-scores derived from bootstrapping ($p_{FDR}$ < 0.05), and the subsequent selection obtained with threshold values of ±3 ended in a PLS+ set including 1474 genes and a PLS− set with 1223 genes. A complete list of the significant genes is provided in Supplementary Materials, Table S1. The highest positive and significant *z*-score was associated with the butyrylcholinesterase (*BCHE*) gene, while the microtubule-associated serine/threonine-protein kinase 4 (*MAST4*) gene represented the opposite extreme. The first gene encodes for an enzyme involved in Aβ binding, while the second gene encodes for proteins with a domain giving the kinase the capacity to shape its own scaffold for controlling its activities (Figure 2, panel B).

### 2.3. Enrichment Analysis

**Figure 3:**
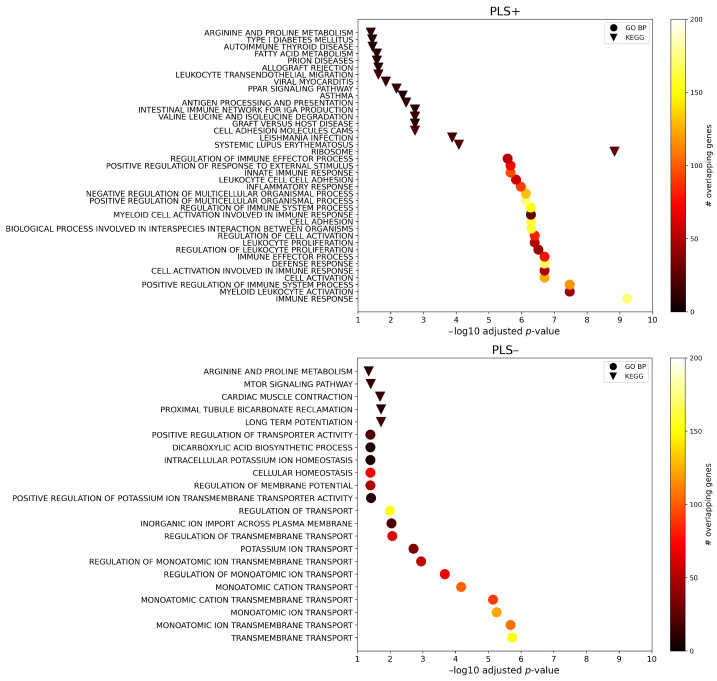
Gene set enrichment analysis of the top genes positively (PLS+, upper part) and negatively (PLS−, bottom part) associated with regional morphometric similarity differences in Aβ+/tau+ and Aβ−/tau− conditions. Overall, only terms with *p*-values surviving the correction for multiple comparisons are shown, and a further selection of the top 20 Gene Ontology biological processes (GO BP) terms is made for PLS+ for the purposes of clearer visualization. The triangles indicate the Kyoto Encyclopaedia of Genes and Genomes (KEGG) pathways, while the circles indicate GO BP terms. The color scale indicates the number of input genes overlapping with the tested gene sets.

Figure 3 shows the gene set enrichment analyses and summarizes the significant Gene Ontology biological processes (GO BP) terms and Kyoto Encyclopaedia of Genes and Genomes (KEGG) pathways ($p_{FDR}$ < 0.05). In general, 363 GO BP terms and 18 KEGG pathways were found for PLS+, whereas 17 GO BP terms and 5 KEGG pathways were found for PLS−. Note that, for ease of visualization, only the top 20 GO BP terms based on the adjusted *p*-value are shown for PLS+ in Figure 3 (a complete list is provided in Supplementary Materials, Table S2). Concerning PLS+, the highest enrichment term based on $p_{FDR}$ was the immune response GO BP ($p_{FDR}$ = $5.94\times 10^{-10}$), which was also the third term overall based on the number of overlapping genes with the input set (=172). Regarding PLS−, the top enrichment term based on the adjusted *p*-value was the transmembrane transport GO BP ($p_{FDR}$ = $1.86\times 10^{-6}$), which was also the second term overall based on the number of overlapping genes with the input set (=149).

**Figure 4:**
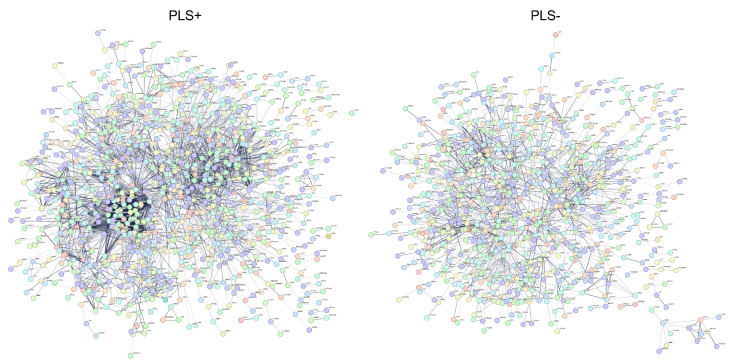
Protein–protein networks from STRING. Maps of interactions between proteins derived from PLS+ (**left**) and PLS− (**right**) sets of genes.

The protein–protein interaction (PPI) network based on the PLS+ gene set resulted in 1360 nodes and 4011 edges, which is 726 interactions more than what would be expected by chance (PPI enrichment *p* < $1.0\times 10^{-16}$). Similarly, the PPI network based on the PLS− gene set resulted in a number of edges significantly higher than the number of interactions expected by chance (PPI enrichment *p* < $1.62\times 10^{-14}$). Indeed, the network had 1176 nodes and 1556 edges, whereas 1275 were expected by chance. A representation of the two networks is provided in Figure 4. The greater presence of clusters is immediately evident in the PLS+-derived PPI network compared to the PLS−-derived PPI network. This was also supported by the higher average node degree and the average local clustering coefficient reached by the first gene set compared to the second one (i.e., 5.90 and 0.413 for PLS+ and 2.65 and 0.356 for PLS−). Additional evidence of the higher level of clusterization of the PPI network built upon the PLS+ gene set was also provided via the detection of significant local clusters, whereas none was found for the PPI network based on the PLS− gene set. In detail, for the PLS+ gene set, the local network cluster with the highest significance was the cluster of the cytoplasmic ribosomal proteins (FDR-corrected *p*-value = $1.43\times 10^{-10}$).

**Figure 5:**
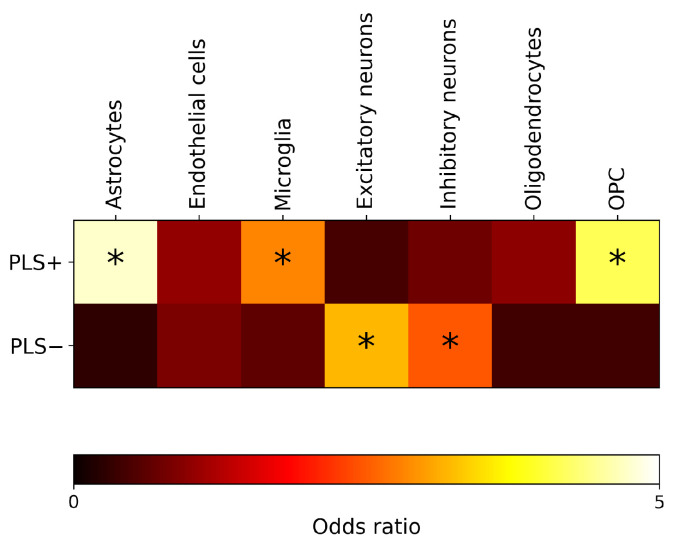
Cell-type enrichment analysis of the top genes positively (PLS+) and negatively (PLS−) associated with regional morphometric similarity differences in Aβ+/tau+ and Aβ−/tau− conditions. The color scale represents the enrichment quantified via odds ratios (ORs), where a high OR indicates high enrichment in genes of a specific cell class. Asterisks denote cell classes for which significant enrichment has been found ($p_{FDR}$ < 0.05). FDR = false discovery rate. OPC = Oligodendrocyte precursors.

Figure 5 shows the results of the enrichment analysis for gene expression in different brain cell types. Statistically significant enrichment ($p_{FDR}$ < 0.05) was found for genes belonging to the PLS+ set, typically expressed in astrocytes, microglia, and oligodendrocytes precursors, while the significantly enriched genes of the PLS− set were those typically expressed in excitatory and inhibitory neurons.

Finally, the enrichment analysis for genes related to AD was found to be significant for the PLS+ gene set (odds ratio, OR = 1.84, *p* = $8.0\times 10^{-4}$) but not for the PLS− one (OR = 0.99, *p* = 0.5). Notably, one of the most common AD-related genes is the Apolipoprotein E (*APOE*) gene, and this was included in the PLS+ gene set.

## 3. Discussion

In this study, we aimed at characterizing the regional MS alterations possibly driven by the brain’s accumulation of Aβ and tau proteins along the AD continuum. Moreover, we showed that such a remodeling is associated with the brain’s expression of genes, which can be related to AD and to Aβ homeostasis. We also highlighted their relationships with cell classes and biological pathways, suggesting possible targets for the assessment of future treatments. To the best of our knowledge, this has not yet been investigated in the current literature, as previous studies on imaging transcriptomics in AD have not focused on biologically defined subtypes nor on assessing the association of brain morphometric changes induced by Aβ and tau accumulation with gene expression.

Despite not surviving the correction for multiple comparisons, the brain regions showing a significant regional MS difference in Aβ+/tau+ compared to Aβ−/tau− revealed a trend of alteration resembling the late stages of amyloid and tau depositions [21]. Braak and Braak [21] examined eighty-three ex vivo brains from dementia and non-dementia subjects and established the neocortex modification as the last two steps of the six indicated for AD neuropathological degeneration. In a more recent work, Grothe et al. [22] confirmed the neuropathological findings by conducting an in vivo staging of the regional amyloid deposition through a multiregional analysis of florbetapir (18F-AV45)–PET data from 667 subjects in the Alzheimer’s Disease Neuroimaging Initiative (ADNI) database. They proposed a four-stage model, according to which, again, the neocortex starts to be involved in the last stages of amyloid progression. Interestingly, by following such a staging model, the caudal anterior cingulate region here found as having a significantly increased MS in Aβ+/tau+ compared to Aβ−/tau− should be the region exposed for the longest time to amyloid deposition. Our work also evidenced significantly increased MS of the lateral occipital cortex in Aβ+/tau+ compared to Aβ−/tau−. Notably, in a very different context from pathology staging, Hwang and colleagues [23] developed an accurate deep generative model to predict the amyloid positivity of CN individuals from structural MR images and other demographic and cognitive information, and the lateral occipital cortex was among the three most relevant regions for the success of the task as disclosed by the integrated gradient explainability method.

The regional MS group-wise differences were correlated, through a PLS fitting, with brain gene expressions as provided by AHBA, resulting in a significant association between the cortical remodeling and the transcription of genes documented to be involved in the late onset of AD [24]. To date, such a phenotype of AD is supposed to be due to many factors, and their precise genetic relationship is still to be fully uncovered [24]. Attempting this goal, genome-wide association studies have identified many susceptibility genes/loci for late-onset AD risk related to Aβ homeostasis [24]. Some of these genes are reported in [24] and are deemed as significant in our work (refer to Supplementary Materials, Table S1). More specifically, in addition to *APOE* and *SORL1*, which are linked to Aβ trafficking, we found *CD33* and *RIN3*, known to be transcripted in the endosomal–lysosomal system for Aβ degradation. Excepting *SORL1*, these genes were significantly expressed in regions with increased MS in Aβ+/tau+ versus Aβ−/tau− individuals. Some of our significantly expressed genes can also be found in studies that specifically focus on phosphorylated tau interactomes (phosphorylated tau is the abnormal state of the protein preceding the neurofibrillary tangles in AD) [25]. Among genes with a positive *z*-score, Drummond et al. [25] showed that *UBA52* encodes for a form of ubiquitin that is one of the most relevant phosphorylated tau interactors. As further confirmation, phosphorylated tau is well known to be ubiquitinated [25]. On the other hand, *HSPA12A* and *PSMD11* highlighted a negative *z*-score in our analysis, and the same genes were found to have highly significant interactions with phosphorylated tau too [25]. The first is considered a novel interactor and is a neuron-specific protein, while the second is related through enrichment to the ubiquitin-proteasome system [25].

Our results show that *BCHE* was the highest expressed gene in regions where MS increased in Aβ+/tau+ compared to Aβ−/tau− (i.e., caudal anterior cingulate, lateral occipital cortex), and it is related to Aβ binding. *BCHE* translates for an enzyme found to be associated to Aβ protein in the final steps of the neuritic plaque maturation process in AD brain tissues [26]. However, in 1997, it was still not known how *BCHE* participates in the process. It was in 2001 that Darvesh et al. [27] demonstrated the interaction of *BCHE* with trypsin, consisting of the enhancement of trypsin catalytic activity depending on *BCHE* concentration. The opposite was not true. This is of interest because serine peptidases similar to trypsin are present in the brain, and the finding suggests that altered *BCHE* may not be able to regulate such catalytic activity anymore, leading to the formation of neuritic plaques.

On the contrary, *MAST4* was the most expressed gene in the regions showing a decreased MS in the Aβ+/tau+ subjects with respect to the Aβ−/tau− individuals (e.g., pars triangularis, posterior cingulate, and pericalcarine). Interestingly, the protein encoded from *MAST4* is a microtubule-associated kinase, whereas tau (from which the neurofibrillary tangles typical in AD originate) is a microtubule-binding protein [20]. Moreover, *MAST4* has been found among the 107 differentially expressed genes in atypical cases of frontotemporal lobar degeneration with fused sarcoma inclusions [28]. Some of the 107 proteins were also associated with other proteomic studies in AD, but *MAST4*, in particular, was shown to be expressed in the prefrontal cortex without knowing the biological pathway to which it belongs. Further comparisons of these proteomic studies with similar ones on AD would be needed to identify proteins and the ways they are related to dementia.

Based on our enrichment analysis, the immune response was the biological process most impacting the regional MS changes shown between Aβ+/tau+ and Aβ−/tau−. Indeed, by focusing on the results obtained for the PLS+ set that was found to be significantly enriched for genes related to AD, many of the processes discovered were associated with the immune response. The immune response has been already documented as being influenced by several genes involved in late-onset AD [24]. For example, the innate immune response was also included among our findings, and it can induce the death of the cells in many different modalities that may lead, in AD, to chronic neuroinflammation linked to the neurodegenerative disease [29]. Strengthening this outcome, our results for the PLS+ set comprised the inflammation response as well. Moreover, microglia and astrocytes are the brain’s immunological cells [30,31], and we observed that they were significantly enriched when looking for PLS+ genes expression in different brain cell types. Single-cell studies have revealed that alterations in inflammatory responses in microglia and astrocytes returned from a complex neuroimmune interplay related to AD pathophysiology [32,33,34].

An additional result from the PLS+ gene enrichment analysis was the involvement of the cytoplasmic ribosomal proteins constituting the most significant cluster found in the corresponding PPI network. Notably, Evans et al. [35] demonstrated the alteration of ribosomal function due to the expression of tau, and particularly in the case of tau mutations due to frontotemporal dementia, as an example of neurodegenerative disease.

The analysis conducted on PLS+ genes further ended in significant enrichment of the oligodendrocyte precursor cell type. Concerning this cell, recently, Zeng and colleagues [36] reported the results from their brain tissue analysis of an AD mouse model exhibiting amyloidosis, tauopathy, and neurodegeneration. These findings were obtained through a new method, namely, STARmap PLUS, combining high-resolution spatial transcriptomics and protein detection. In their work, Zeng et al. unexpectedly observed that oligodendrocyte precursors were enriched at an intermediate spatio-temporal distance from the appearance of the Aβ plaques. Such an enrichment potentially subtends localized proliferation and differentiation of the oligodendrocyte precursors to mature oligodendrocytes. The authors of [36] suggested that many layers arise and surround the plaques, and oligodendrocyte precursors and oligodendrocytes may constitute one of the last layers after those composed of reactive microglia.

On the other hand, the PLS− set did not show any significant local clusters in the derived PPI network, and it was not significantly enriched for genes related to AD, even though we found significant enrichment for biological processes related to transmembrane transport and for both excitatory and inhibitory neuron cell types. This may be linked to the main limitation of our work, consisting in the low number of subjects retained in our cohort. Indeed, a higher number of individuals would probably give greater statistical power to the cortical MS patterns captured in this study, also representing a cascade of benefits for the imaging transcriptomics associations disclosed. In addition, we included MCI patients in the control cohort to increase the sample size and better match the clinical severity of the Aβ+/tau+ group. However, a subset of MCI patients with Aβ−/tau− status may exhibit different pathophysiological mechanisms. Further research is needed to assess the influence of these individuals on the imaging transcriptomics relationship. However, in an attempt to control these issues, we maintained a balanced numerosity of the two groups under investigation, and we used the Mini-Mental State Examination (MMSE) as the covariate. Future works will increase the resolution of the investigation by including Aβ+/tau− and Aβ−/tau+ groups. This will entail the uncovering of distinct mechanisms underlying the two pathways, perhaps providing support for understanding the different theories underlying AD [1].

## 4. Materials and Methods

### 4.1. Data Cohort

The data used in the preparation of this article were obtained from the ADNI database (adni.loni.usc.edu). The ADNI was launched in 2003 as a public–private partnership, led by Principal Investigator Michael W. Weiner, MD. The primary goal of ADNI has been to test whether serial MRI, PET, other biological markers, and clinical and neuropsychological assessment can be combined to measure the progression of MCI and early AD.

A total of 298 subjects from ADNI-3 were selected among those diagnosed as CN, significant memory concern (SMC), and MCI (Stages I–III), excluding patients with dementia (Stages IV–VI) [37], in order to selectively capture Aβ and tau transcriptomic signatures in AD pre-clinical conditions. Of note, when the present study was initiated, ADNI-4 was just starting to enroll participants, and their number was negligible for this investigation. ADNI is IRB-approved, and all participants signed their informed consent. For every subject, we collected a 3D T1w-MR image and single-shell dMRI volumes along with information on age, gender, years of education, MMSE score, and concentrations of Aβ and tau protein in the cerebrospinal fluid. All these participants had their MRI taken and their Aβ and tau concentrations collected within 12 months from each other.

Concerning MRI, the following acquisition parameters were used: (i) 3D T1w-MRI: sagittal accelerated MPRAGE, TR/TE = shortest, TI = 900 ms, FOV = 208 × 240 × 256 mm^3^, flip angle = 9°, resolution = 1 × 1 × 1 mm^3^; (ii) dMRI: TR/TE = 7200/56 ms, FOV = 232 × 232 × 160 mm^3^, resolution = 2 × 2 × 2 mm^3^, *b* = 0 and 1000 s/mm^2^. Note that, while the dMRI protocol of ADNI-3 includes both single- and multi-shell acquisitions, only the former was considered here [38].

The subjects were categorized as Aβ−/tau− if [Aβ] ≥ 980 pg/mL and [tau] ≤ 24 pg/mL and vice versa for Aβ+/tau+ [39].

Table 1 summarizes the main sociodemographic and clinical information about the cohort under investigation.

### 4.2. Magnetic Resonance Imaging Data Processing

The T1w-MR images were minimally preprocessed by using *fsl_anat* tool (FSL, version 6.0, https://fsl.fmrib.ox.ac.uk/fsl/fslwiki/ (accessed on 25 November 2024)) [40] to correct for bias field artifacts and to linearly register them to the 2 mm MNI space. FreeSurfer (version 7.0, https://surfer.nmr.mgh.harvard.edu/ (accessed on 25 November 2024)) [41] was then employed to extract 84 regions of interest (ROIs) for each subject by referring to the Desikan–Killiany atlas. Subcortical ROIs were discarded together with ROIs belonging to the right hemisphere for coherence of analysis, with the data coming from AHBA (http://human.brain-map.org/ (accessed on 25 November 2024)) [42]. Please refer to Section 4.4 for details. Then, 7 different morphometric descriptors were considered for the 34 cortical ROIs in the left hemisphere. These included area, fold index, curvature index, Gaussian curvature, mean curvature, thickness, and volume.

FSL [43] was used to extract the brain and to correct for Eddy current artifacts in the dMRI data. The Python *dipy* library (https://dipy.org/ (accessed on 25 November 2024)) [44] was subsequently employed to denoise data by performing a principal component analysis and by automatically classifying the partial components relying on the Marcenko–Pastur distribution (3D sliding window radius set to 2) [45]. For each subject, the dMRI volumes corresponding to *b* = 0 were averaged, the resulting image was registered to the T1w volume of the subject by taking advantage of the *epi_reg* routine in FSL [46], and it followed a linear alignment to the MNI space thanks to the *fsl_anat*-derived transformation. Then, all the dMRI volumes were linearly transformed by applying the transformations obtained from the average *b0* processing. Finally, the result obtained for each subject was corrected for EPI-induced currents through a non-linear registration to the MNI space [47] with ANTs software (version 2.3, http://stnava.github.io/ANTs/ (accessed on 25 November 2024)) [48], and the direction of the gradients was rotated accordingly. The diffusion tensor model [49] in the *dipy* library was used to derive the standard mean diffusivity, radial diffusivity, fractional anisotropy, and the three diffusion tensor’s eigenvalue microstructural maps [49]. Finally, the indices’ mean value for each of the aforementioned 34 ROIs was calculated.

### 4.3. Regional Morphometric Similarity Group-Wise Differences

For each subject, the 13 features calculated for each of the 34 ROIs, including both morphometric measures from T1w images and microstructural descriptors from dMRI data, were used to construct the individual MSN. More specifically, all the features were separately normalized by subtracting the mean and dividing by the standard deviation across all ROIs. Pearson’s correlation coefficient was then calculated for each pair of ROIs, leading to a subject-specific MSN (i.e., a 34 × 34 symmetric matrix). Each regional MS was thus computed by calculating the sum of the absolute value of all the correlation coefficients obtained for the considered ROI with all the other ROIs and dividing by the number of all the ROIs except for the one considered.

The differences in regional MS between the Aβ+/tau+ and Aβ−/tau− groups were assessed by fitting, for each ROI, a linear model considering the sociodemographic and clinical information described in Table 1 as covariates. *t*-statistics were used as variables for the analysis detailed in Section 4.5, while *p*-values were adjusted for multiple comparisons through a FDR threshold (<0.05). The analyses were performed in Python, taking advantage of the *statsmodels* (https://www.statsmodels.org/stable/ (accessed on 25 November 2024)) and *SciPy* packages (https://docs.scipy.org/doc/scipy-1.14.0/ (accessed on 25 November 2024)).

### 4.4. Gene Expression: Allen Human Brain Atlas

The AHBA dataset includes post-mortem brain-wide gene expression data from 6 donors [age = 42.5 (13.38) years, male/female = 5/1] [42]. The data were processed and mapped to 84 parcellated brain regions from the Desikan–Killiany atlas by employing the *abagen* toolbox (https://github.com/netneurolab/abagen (accessed on 25 November 2024)) [50], but only the left hemisphere (i.e., 34 ROIs) was considered in the current work due to the fact that only two of the six donors effectively presented the right hemisphere [51]. The reannotation of the microarray probes was performed by using data from [51] and discarding those not matching a valid Entrez ID. The probes were subsequently filtered based on the intensity value of their expression relative to the background noise. When more than one probe was related to the expression of the same gene, the probe’s selection was based on the differential stability criterion, which points at the probe showing a pattern of regional variation mostly consistent across the 6 subjects. These steps led to 15633 probes, each one corresponding to a single gene. Then, based on the tissue sample MNI coordinates, the brain region corresponding to each sample was identified as the nearest within a 2 mm radius. In the event that no match was found, the nearest sample to the centroid of the region was chosen. The average value for each sample assigned to the same brain region was calculated for every donor. Then, the values of the gene expression were normalized separately for each of the 6 subjects over the regions by relying on a robust sigmoid function and rescaling to the unit. Finally, the gene expression values were averaged across donors ending in a resultant matrix of dimensions 34 × 15633 (i.e., ROIs × genes).

### 4.5. Regional Morphometric Similarity and Transcriptomics Relationship

The Aβ+/tau+ and Aβ−/tau− differences in regional MS, represented by the *t*-statistics previously calculated, were associated with the transcriptomics data through a PLS regression [13]. More specifically, the regional MS differences represented the response variables predicted by the measurements of the gene expression (i.e., the predictors). PLS regression allows us to find the best linear combination of gene expression values based on the maximum covariance they present with the regional MS variations induced by Aβ/tau conditions. The PLS regression was performed by using the Python *scikit-learn* package (https://scikit-learn.org/stable/ (accessed on 25 November 2024)) and specifying one latent component to search for after the standardization of the predictors. The PLS model’s significance was then assessed through a permutation test performed by permuting the response variables $10^{4}$ times [52].

The contribution of each gene in the PLS model was calculated by bootstrapping [13]. In detail, such a contribution was quantified based on the *z*-score, calculated for every gene as the ratio of the corresponding PLS weight and the standard error obtained by resampling the 34 ROIs with replacements. Highly positive *z*-scores correspond to genes with a great positive contribution to PLS, meaning that they are overexpressed in ROIs with increased MS. Conversely, highly negative *z*-scores identify the genes with a great negative contribution to PLS that translates to an overexpression in ROIs with decreased MS. The genes were then ranked according to their *z*-score and selected after the FDR correction based on the *p*-value ($p_{FDR}$ < 0.05). A further selection was made by deriving the list of genes with *z* > 3 (PLS+) and the list of genes with *z* < -3 (PLS−). Both lists underwent an enrichment analysis as described in the following section.

### 4.6. Enrichment Analysis

The *GENE2FUNC* tool of the Functional Mapping and Annotation of Genome-Wide Association Studies (FUMA GWAS, https://fuma.ctglab.nl/ (accessed on 25 November 2024)) platform [53] was used to investigate the GO and KEGG databases to find the pathways and the BP involving PLS+ and PLS− genes, respectively. The background genes used were those belonging to the final list coming from the processing described in Section 4.4 (i.e., 15633 genes), and the results of the gene set enrichments were FDR-corrected.

STRING (version 12.0, https://string-db.org/ (accessed on 25 November 2024)) [54] was employed to build the PLS+ and the PLS− protein–protein functional interaction networks. We did not include text mining among the possible active interaction sources, and we set the minimum required interaction score to the default (i.e., 0.400).

To investigate the enrichment of PLS+ and PLS− genes for specific types of brain cells, as in [55], we compiled the data coming from 5 single-cell studies employing postmortem cortical samples in human postnatal subjects [56,57,58,59,60]. All the 5 study-specific gene lists were merged by cell type, and we avoided duplicates and removed genes not present in the AHBA dataset. In detail, the neuron-related gene sets from [56,60] were discarded because the authors did not provide any specific distinction between the excitatory and inhibitory neurons as the others did, while the gene set related to pericyte was excluded because such a cell type was considered only in [58]. The whole process ended in 7 gene sets corresponding to genes specifically expressed in microglia, endothelial cells, oligodendrocyte precursors, oligodendrocytes, astrocytes, excitatory and inhibitory neurons. The cell-class enrichment analyses were performed in R, using the *GeneOverlap* package (version 1.40.0) [61]. The same background used in the enrichment analysis and consisting of the 15633 AHBA genes was kept unaltered for this analysis. The overlap between the individual PLS+ and PLS− gene sets and each of the 7 cell-specific gene sets was tested through Fisher’s exact test. The FDR correction for the number of cell types was applied to identify significant overlaps ($p_{FDR}$ < 0.05). Finding a significant overlap means that the overlap between the two sets of genes is greater than the overlap that can be obtained with a random selection of a set of the same number of genes from the background.

Finally, *GeneOverlap* was also used to inspect the enrichment of PLS+ and PLS− genes for AD-related genes. The sources for defining such an AD-related genes list were (i) all the genes having loci with genetic evidence and all the AD risk/protective causal genes as compiled by the AD sequencing project gene verification committee and cleaned of duplicates (https://adsp.niagads.org/gvc-top-hits-list/ (accessed on 25 November 2024)); (ii) all the AD-associated genes retrieved in [62] through the employment of HETIONET (https://het.io/ (accessed on 25 November 2024)); (iii) and the genes involved in AD collected in DisGeNET (https://disgenet.com/ (accessed on 25 November 2024)) [63]. The three lists were merged, duplicates were avoided, and those genes that were not present among the 15633 AHBA genes used in this study were discarded, leading to a total number of 245 AD-related genes. PLS+ and PLS− gene sets were individually tested for overlapping with such a gene set by specifying the 15633 AHBA genes as the background.

## 5. Conclusions

Regional MS differences between Aβ+/tau+ and Aβ−/tau− along the AD continuum were assessed in this work for the first time. The alterations correlating with gene expression in the brain as provided by AHBA, resulting in *BCHE*, a gene involved in Aβ binding, had the most impact. Moreover, the enrichment analysis suggested that the immune response, astrocytes, microglia, and oligodendrocyte precursors are the biological process and cell types possibly leading to the cortical MS remodeling experienced by individuals before AD diagnosis when the Aβ/tau status changes from negative to positive.

## Figures and Tables

**Table 1:** Demographic, social, and clinical details of the study cohort. The mean value and the standard deviation [mean (SD)] are reported for age and education years (Y) and mini-mental state examination (MMSE) score. Gender is reported as count of males over females (M/F). The *p*-value was retrieved from the Mann–Whitney non-parametric U-test for age, education, and MMSE, and from the $\chi^{2}$ test for gender.

Covariate	Aβ−/tau−	Aβ+/tau+	*p*-Value
Age [Y]	71.05 (7.10)	77.18 (7.92)	$4\times 10^{-11}$
Gender [M/F]	71/101	60/66	0.148
Education [Y]	16.70 (2.38)	16.01 (2.54)	0.016
MMSE	28.85 (1.47)	26.06 (4.62)	$2\times 10^{-11}$

## Data Availability

The data employed in this work were obtained from a third-party organization: ADNI. All ADNI data are shared through the LONI Image and Data Archive (IDA), a secure research data repository. Interested scientists may obtain access to ADNI imaging, clinical, genomic, and biomarker data for the purposes of scientific investigation, teaching, or planning clinical research studies. Access is contingent on adherence to the ADNI Data Use Agreement and the publication policies outlined in the documents listed at the following link: https://adni.loni.usc.edu/.

## Supplementary Materials

The following supporting information can be downloaded at https://www.mdpi.com/article/10.3390/ijms1010000/s1.

## Abbreviations

The following abbreviations are used in this manuscript:

MRI	Magnetic resonance imaging
PET	Positron emission tomography
Aβ	Amyloid-β
AD	Alzheimer’s disease
T1w	T1-weighted
dMRI	Diffusion MRI
AHBA	Allen Human Brain Atlas
MS	Morphometric similarity
CN	Cognitively unimpaired
MCI	Mildly cognitively impaired
MSN	MS network
SMC	Significant memory concern
Aβ+/tau+	Aβ-positive/tau-positive
Aβ−/tau−	Aβ-negative/tau-negative
ADNI	AD Neuroimaging Initiative
MMSE	Mini-mental state examination
ROI	Region of interest
FDR	False discovery rate
PLS	Partial least squares
FUMA GWAS	Functional Mapping and Annotation of Genome-Wide Association Studies
KEGG	Kyoto Encyclopedia of Genes and Genomes
GO BP	Gene Ontology biological process
PPI	Protein–protein interaction
OR	Odds ratio
